# Recruitment difficulties in a primary care cluster randomised trial: investigating factors contributing to general practitioners' recruitment of patients

**DOI:** 10.1186/1471-2288-11-35

**Published:** 2011-03-31

**Authors:** Matthew J Page, Simon D French, Joanne E McKenzie, Denise A O'Connor, Sally E Green

**Affiliations:** 1 School of Public Health and Preventive Medicine, Monash University, Melbourne, Australia; 2 Primary Care Research Unit, University of Melbourne, Melbourne, Australia

## Abstract

**Background:**

Recruitment of patients by health professionals is reported as one of the most challenging steps when undertaking studies in primary care settings. Numerous investigations of the barriers to patient recruitment in trials which recruit patients to receive an intervention have been published. However, we are not aware of any studies that have reported on the recruitment barriers as perceived by health professionals to recruiting patients into cluster randomised trials where patients do not directly receive an intervention. This particular subtype of cluster trial is commonly termed a professional-cluster trial. The aim of this study was to investigate factors that contributed to general practitioners recruitment of patients in a professional-cluster trial which evaluated the effectiveness of an intervention to increase general practitioners adherence to a clinical practice guideline for acute low-back pain.

**Method:**

General practitioners enrolled in the study were posted a questionnaire, consisting of quantitative items and an open-ended question, to assess possible reasons for poor patient recruitment. Descriptive statistics were used to summarise quantitative items and responses to the open-ended question were coded into categories.

**Results:**

Seventy-nine general practitioners completed at least one item (79/94 = 84%), representing 68 practices (85% practice response rate), and 44 provided a response to the open-ended question. General practitioners recalled inviting a median of two patients with acute low-back pain to participate in the trial over a seven-month period; they reported that they intended to recruit patients, but forgot to approach patients to participate; and they did not perceive that patients had a strong interest or disinterest in participating. Additional open-ended comments were generally consistent with the quantitative data.

**Conclusion:**

A number of barriers to the recruitment of patients with acute low-back pain by general practitioners in a professional-cluster trial were identified. These barriers were similar to those that have been identified in the literature surrounding the recruitment of patients in individual patient randomised trials. To advance the evidence base for patient recruitment strategies in primary care settings, trialists undertaking professional-cluster trials need to develop and evaluate patient recruitment strategies that minimise the efforts required by practice staff to recruit patients, while also meeting privacy and ethical responsibilities and minimising the risk of selection bias.

**Trial registration:**

Australian New Zealand Clinical Trials Registry ACTRN012606000098538 (date registered 14/03/2006).

## Background

Patient recruitment is often reported as the most challenging step in conducting randomised trials (RTs) and data on effective recruitment strategies is lacking [1-3]. This is particularly the case for cluster randomised trials (CRTs), where intact social groups (for example, primary care clinics or schools), rather than individuals, are the unit of randomisation [4,5]. Lower level evidence in the form of case studies describing recruitment successes and failures experienced in individual patient RTs [6-10] and CRTs which recruit patients to receive an intervention [11-13] exists. Results of these studies suggest that barriers such as time constraints, forgetting to recruit, and too few eligible patients influence recruitment rates [9,14,15]. However, to our knowledge, no studies have been undertaken to investigate factors associated with recruitment of patient participants for a particular subtype of CRT known as a professional-cluster trial [16]. In these trials, interventions are targeted at health professionals, and while patients are likely to be effected by any change in health professional behaviour, patients do not directly receive an intervention. The paucity of research for this subtype of CRT is perhaps unsurprising, since it is preferable to have separation in cluster trials between those receiving the intervention (and thus aware of their allocation), and those recruiting patient participants [17]. However, there are instances where due to privacy issues, and financial and feasibility constraints, it becomes difficult to have this separation. Different barriers to recruitment of patient participants in this type of trial may exist compared with other designs. Therefore, the aim of this study was to investigate factors that contribute to GPs' recruitment of patients in a professional-cluster trial.

Professional-cluster trials are frequently used in the field of implementation science to evaluate interventions designed to increase uptake of research into clinical practice [18,19]. In these trials, patients may be recruited to provide measures of patient outcomes (for example, health outcomes), or measures of practitioner behaviour (for example, whether their GP provided a particular treatment), or both. The latter may occur in instances where it is not possible to measure practitioner behaviour through other sources (for example, clinical records or administrative data).

Recruitment of patient participants into professional-cluster trials can result in selection bias from the selective recruitment of patients by individuals who are often aware of intervention allocation (for example, Farrin 2005 [11]). Strategies to minimise the risk of this selection bias in professional-cluster trials have been recommended, such as identification of patients prior to treatment allocation, blinding of recruiters, and collection of routine data available in patient files [20,21]. In Australia, collection of data from patient medical files can only be undertaken with the patient's consent for a particular study [22]. Further, health professionals cannot provide researchers with contact details of patients to invite them to give this consent without the patient's permission. This poses a number of challenges to trialists undertaking professional-cluster trials in Australian primary care settings, as the privacy and ethical responsibilities, resources available, and the nature of the clinical condition means it may only be possible to recruit patients through practitioners who are aware of their allocation status.

While it may be supposed that the factors that contribute to health professionals' patient recruitment rates in professional-cluster trials are similar to those in individual patient RTs, differences may also be expected. For example, in professional-cluster trials, patients are less likely to perceive they will directly gain, and so may be less interested in being involved, or their health professional may perceive this to be the case and be less motivated to approach them for participation [14,23]. Determining whether this poses a barrier to patient recruitment is useful as it suggests that the way to promote participation may have to be more targeted for professional-cluster trials. The IMPLEMENT CRT [24] provided an opportunity to explore factors contributing to poor patient recruitment in a professional-cluster trial.

### Design of the IMPLEMENT CRT

The IMPLEMENT CRT was funded by the Australian National Health and Medical Research Council (NHMRC) to test the effectiveness of a theory-based intervention to implement a clinical practice guideline (CPG) for acute non-specific low-back pain (LBP) into general practice in Victoria, Australia [24]. Forty-five primary care practices (59 GPs) were randomly allocated to the intervention, comprising two interactive workshops incorporating behaviour change techniques to target barriers to CPG implementation, and 47 practices (53 GPs) were allocated to the control arm of dissemination of a printed copy of the CPG. The primary GP-level outcome was whether the GP referred the patient for a plain x-ray within three months post-patient enrolment determined from medical records of consenting patients. The primary patient-level outcome was LBP-specific disability three months post-enrolment, collected via telephone interviews. Details of secondary outcomes and eligibility criteria for general practices, GPs, and patients are available in the protocol [24]. We aimed to recruit 2300 patient participants from 92 practices.

### Recruitment strategies used in the IMPLEMENT CRT

**Figure 1:**
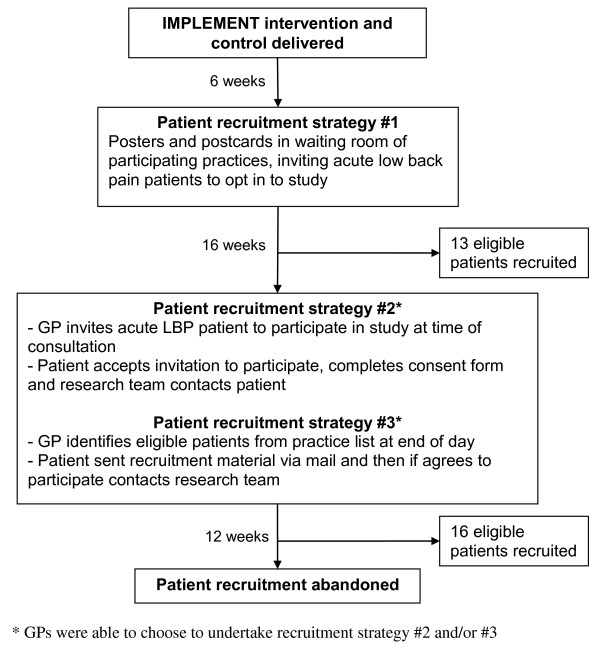
**Patient recruitment process for the IMPLEMENT cluster randomised trial**.

Three recruitment strategies were used during the CRT. No financial incentives were offered to the GPs to recruit patients and GPs were not affiliated with the academic centre that conducted the study. The first strategy was designed to minimise potential selection bias from selective recruitment of patients and did not involve GPs identifying potential participants. Recruitment strategies two and three were more susceptible to selective recruitment since in these strategies the GPs were involved in identifying potential participants. These recruitment strategies, and the numbers of participants recruited, are outlined in Figure 1.

### Aim

The reasons for the poor response to recruitment strategies two and three were unclear and raised several questions. Did the GPs disseminate the recruitment handouts? Were approached patients interested? Did GPs have difficulty recruiting patients? What were the reasons for this? The aim of this study was therefore to investigate factors that contributed to GPs' recruitment of patients with acute LBP in the IMPLEMENT CRT.

## Methods

Nine months after patient recruitment began, a questionnaire was mailed to all GPs still participating in the IMPLEMENT CRT (94 GPs from 81 practices). Eleven practices (six from the intervention group and five from the control group) comprising 18 GPs dropped out of the study prior to the start of patient recruitment, so were not posted a questionnaire. GP reasons for withdrawing from the trial before patient recruitment included being too busy to continue, GP left the included practice and GP poor health. Non-responders to this questionnaire were contacted by mail reminders and by telephone.

**Figure 2:**
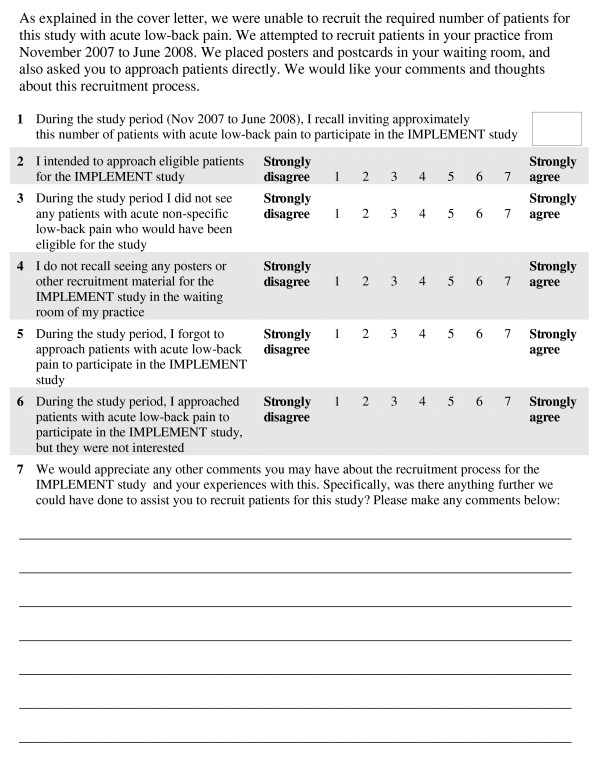
**General practitioner questionnaire about patient recruitment**.

This questionnaire included seven items to assess possible reasons for the poor recruitment of patients as perceived by the GPs (see Figure 2). The authors developed the questionnaire by creating an item for each of the hypothesised reasons for poor recruitment in the trial, and then piloted the items with the clinical investigators. One item asked GPs to recall the number of patients with acute LBP they invited to participate over the recruitment period. Five seven-point Likert-scale items were used to measure factors which were potentially predictive of recruitment (for example, intention to recruit and forgetting to recruit). An open-ended item was included for GPs to provide comments about the recruitment process and whether there was anything further they thought we could have done to assist them. Double data entry was used to enter the questionnaire data, and discrepancies were resolved via discussion with a third researcher. Descriptive statistics (means, medians, inter-quartile ranges (IQRs) and 95% confidence intervals (CIs)) were calculated for the quantitative items, based on the available data for each item. Data were analysed at the practice level to adjust for correlation that may occur between GPs within the same practice by calculating an average response for practices with more than one GP (9/68 practices).

Comments provided in response to the open-ended question that were interpreted as being sufficiently similar in theme were coded into categories developed by two researchers independently. Coding of comments provided in response to the open-ended question was done freely rather than based on a pre-developed framework. Two researchers independently extracted themes from each comment, for example, if a GP wrote "I am sorry but I just forgot to recruit patients for this study", a possible theme nominated could be 'Forgot to recruit patients'. The researchers listed more than one theme/category resulting from each comment if applicable. Any discrepancies in the themes assigned to each comment by the two researchers were resolved via discussion.

Ethical approval for this study was obtained from the Monash University Standing Committee on Ethics in Research involving Humans (2006/047).

## Results

Seventy-nine GPs completed at least one item (79/94 = 84% GP response rate), representing 68 of 81 practices (84% practice response rate) who participated in the patient recruitment strategies. Just under half the GPs (n = 44) provided a response to the open-ended question. Table 1 shows the baseline characteristics of those GPs who were posted the survey about patient recruitment, and those who dropped out of the trial before the patient recruitment phase. Most characteristics were similar, except that for those who dropped out of the study, none of them reported a special interest in LBP, compared to 17% of those GPs who stayed in the study and were posted the final questionnaire.

**Table 1:** General practice and general practitioner baseline characteristics

	Participated in patient recruitment and posted questionnaire		Dropped out of CRT prior to patient recruitment	
**Practice factors at baseline**	*N practices*		*N practices*	
Number of practices	81		11	
Number of GPs per practice (SD)	81	5 (3.8)	11	6 (4.1)
No. (%) rural practices	81	29 (36)	11	2 (18)
No. (%) with x-ray facility on site	80	3 (4)	9	1 (11)
No. (%) of industrial practices	81	7 (9)	9	0 (0)
No. (%) of training practices	80	51 (64)	9	6 (67)
Method of billing:	76		9	
No. (%) Bulk bill		12 (16)		1 (11)
No. (%) Co-payment		64 (84)		8 (89)
**GP factors at baseline**	*N GPs*		*N GPs*	
Number	94		18	
Mean age (years) (SD)	93	54 (10.3)	8	55 (14.1)
No. (%) female	93	33 (35)	10	4 (40)
Mean number of years since graduated (SD)	93	30 (10.2)	8	31 (14.5)
No. (%) with special interest in low-back pain	93	16 (17)	8	0 (0)
No. (%) undertaken LBP continuing education in past year	92	10 (11)	8	1 (12)
Mean number of patients seen per week (SD)	92	126 (55.4)	8	129 (61.6)
Mean number of LBP patients seen per week (SD) (averaged over the previous month) [Median; IQR]	90	12 (17.7) [8; 4 to 10]	8	11 (5.4) [10; 9 to 12]
No. (%) who are members of local GP Division	93	87 (93)	8	7 (87)

CRT = Cluster randomised trial; SD = standard deviation; GPs = general practitioners; IQR = Interquartile range [25^th ^percentile - 75^th ^percentile]

The sample of respondents consisted of GPs with a mean age of 52.2 years (SD = 11.2), who practised on average 38.6 hours per week (SD = 13.4), 52 were males (66%) and 39 GPs were allocated to the intervention. When surveyed at baseline, the GPs reported consulting a median of eight patients with acute LBP per week (IQR 4-10).

General practitioners recalled inviting a median of two patients with acute LBP (interquartile range 0 - 5.5) to participate in the IMPLEMENT CRT, with a maximum of 20 patients. Twenty-six GPs (28%) reported inviting no patients. Across the 71 GPs who responded to this item, the total number of patients they recalled inviting during the recruitment period was 287.

Table 2 describes GPs' responses to patient recruitment items in the survey. GPs had high intention to approach eligible patients with acute LBP into the trial (item 2), and they indicated they did treat some patients with acute LBP during the recruitment period (item 3). On average, GPs recalled some awareness of seeing the recruitment material for the trial in their waiting rooms (item 4); however, there was some variability in this awareness. GPs indicated they forgot to approach patients with acute LBP to participate in the trial (question 5), but when they did approach patients to participate, patients showed neither an interest nor disinterest in participating (question 6).

**Table 2:** General practitioners' responses to patient recruitment items in the survey

Survey item*	*n (practices)*	*Mean*	*95% CI*	*Median*	*IQR*
GP intended to invite eligible patients to participate in the study	67	5.6	5.2 - 5.9	6	5 - 6.5
GP did not see eligible patients during the study period	66	2.7	2.3 - 3.1	2	1 - 3.9
GP did not recall seeing patient recruitment materials in the practice	66	3.1	2.7 - 3.6	3	1.6 - 4
GP forgot to approach eligible patients to participate in the study	67	4.4	4.0 - 4.8	5	3.3 - 6
GP approached patients to participate, but they were not interested	67	3.5	3.1 - 3.9	4	2 - 5

* Scored on a 7-point Likert scale from strongly disagree (1) to strongly agree (7)

Analysis of the open-ended comments identified six reasons for the poor recruitment: time constraints, few eligible patients, forgot to recruit patients, confusion about recruitment strategies, lack of patient interest and lack of patient incentives (Table 3). There were nine coding discrepancies, out of a total 44, between the two coders that were all resolved via discussion.

**Table 3:** Main themes identified from open-ended question, with illustrative quotes

Theme	Illustrative quote
Time constraints	"I intended to try to recruit patients but I think there is too much going on during the consultation to assess LBP that to add to it by discussing a 'study' would have been information overload. So unfortunately, recruiting for your study was the first thing to go - just not enough time for everything in general practice."
Few eligible patients	"Most of my patients have chronic LBP and were not eligible for the study. Only a couple I encountered who were eligible."
Forgot to recruit patients	"It probably would have helped to have a few reminder emails. I tried to make myself remember by putting up a sign at eye level beside my computer, but that didn't help."
Confusion about recruitment strategies	"All the documentation and stamps arrived. My understanding was that your researcher or office would contact our office manager to explain the study. This did not occur. I was left without direction."
Lack of patient interest	"Few patients were approached and not interested, then I lost the interest."
Lack of patient incentives	"Suggest a free physiotherapy appointment/or better still a $30 petrol voucher for their time if complete survey. You would be inundated."

## Discussion

The results of this survey provide some insight into factors that contributed to recruitment issues in the IMPLEMENT professional-cluster trial. The results suggest that few patients with acute LBP were invited to participate in the trial, despite GPs indicating at the beginning of the study that they saw a median of eight eligible patients per week on average. Despite intending to recruit patients, GPs reported forgetting to approach patients to participate, and did not perceive that patients had neither a strong interest nor disinterest in participating. Additional open-ended comments were coded into six categories that were generally consistent with the quantitative data.

Failure to recruit the target number of patients in trials in the primary care setting is common [25]. Frequently reported barriers in RTs and CRTs where patients receive an intervention include time constraints, forgetting to recruit, and few eligible patients treated [9,14,15]. Results from the current study suggest that similar recruitment barriers may be faced in professional-cluster trials. Our hypothesis that lack of an intervention being directly targeted at patient participants may pose a major barrier to GPs recruitment of patients was not supported by the data. With the large number of clinical tasks GPs conduct during a consultation, it is likely that even if patients were very interested in participating, and GPs perceived this to be the case, the chances of them having limited time to invite patients into the study, or forgetting to do this, were probably high. Given the identified barriers in this study were similar to those reported in individual patient RTs, interventions which are effective in enhancing patient recruitment in individual patient RTs may also be applicable in professional-cluster trials.

This study highlights an important challenge for trialists planning to recruit patients in professional-cluster trials undertaken in Australian primary care; namely, minimising the impact of the trial on practice staff time, maximizing the recruitment rate, and minimising the chance of selection bias. The use of advertisements in the waiting rooms during the first phase of recruitment in the IMPLEMENT CRT was designed to minimise this risk of selection bias arising from GPs recruiting patients and to reduce the burden on GPs and practice staff. On average, the GPs recalled seeing these advertisements in their practice, but such a passive strategy was not successful. The second two recruitment strategies had a high possibility of selective recruitment of patients, and in addition, required a greater time commitment from GPs and practice staff, and were also unsuccessful. Use of alternate strategies to minimise selection bias were not possible given available resources. To prevent bias from occurring in future professional-cluster trials, the ethics and feasibility of alternative methods of obtaining patient data other than direct approach of patients needs to be considered [19].

### Methods for evaluating recruitment strategies

A number of different methods exist to evaluate patient recruitment strategies. The most rigorous method is a RT of different recruitment methods nested within a 'host' RT. When conducted properly this enables the recruitment rates to each strategy to be attributed to the recruitment strategy [26]. However, nested RTs pose a number of methodological challenges, including increased management burden, preferences of people recruiting participants for one recruitment strategy over another, and the potential impact on the statistical power of the 'host' RT [26]. Alternate methods of evaluating recruitment strategies, including recruitment barrier surveys, are commonly undertaken following the failure to recruit patients. However, many of these observational studies are of poor quality. For example, a review of recruitment barrier studies in cancer RTs identified 27 studies using such surveys, many of which only included one item, providing limited data on reasons for poor recruitment [27]. Further, many surveys did not provide respondents with an opportunity to make additional comments, which can result in potentially valuable, unexpected, data being missed and limit potential hypothesis generation of reasons for poor recruitment. Additionally, response rates of these studies were infrequently reported, making it difficult to determine the extent to which the data reported is at risk of selection bias [27]. To advance the evidence base, along with conducting high quality nested recruitment RTs, more care needs to be taken in the design of observational studies evaluating barriers to patient recruitment strategies.

### Limitations

The questionnaire only comprised seven items, which limits the amount of information provided. However, when developing the questionnaire our intention was to minimise responder burden, and the response rate of 84% to the six quantitative items suggests that health professionals are likely to complete a relatively short questionnaire. However the 16% non-responders may have provided different information about recruitment that may have affected our results. Second, all items may have been influenced by social desirability bias, the tendency of respondents to want to appear in a positive light [28]. Responses to all items were also dependent on the GPs' recall of the patient recruitment period which started nine months prior to receiving the questionnaire. Determining the test-retest reliability of the items may have decreased the measurement error associated with responses [29]. Another limitation, and one that is shared with many surveys, is that the content of the open-ended comments may have been influenced by the preceding items. Finally, the process of coding the open-ended comments necessarily involved some subjectivity and may have been biased by coders' expectations about the reasons for poor patient recruitment. To limit this subjectivity, two researchers coded the comments independently and all disagreements, of which there were few, were resolved via discussion. However it is still possible that other researchers may have coded the comments differently.

## Conclusions

A brief questionnaire provided some insight into GPs perceptions about the reasons for the failure to recruit patients with acute LBP into a professional-cluster trial. The barriers to patient recruitment identified in this study were similar to those reported in trials which recruit patients to receive an intervention. Results indicate that while GPs intended to recruit patients, barriers such as time constraints and forgetting to recruit patients may have contributed to poor recruitment. To advance the evidence base for patient recruitment strategies in professional-cluster trials in the primary care setting, trialists need to develop and evaluate patient recruitment strategies that minimise the efforts required by practice staff to recruit patients, meet privacy and ethical responsibilities, while also minimising the risk of recruiting a selective sample of patients.

## Competing interests

The authors declare that they have no competing interests.

## Authors' contributions

SEG, JEM, DAO and SDF conceptualized and designed the IMPLEMENT CRT and secured funding. SEG was the lead investigator of the funding application. MJP provided input on the recruitment strategies used in the CRT. SDF and SEG conceived the study to measure reasons for poor patient recruitment in the IMPLEMENT CRT and developed the questionnaire. MJP and SDF collected and analysed the recruitment questionnaire data. MJP wrote the first draft of the publication. JEM suggested some conceptual changes to the manuscript and contributed sections of text in the Background, Methods, Results and Discussion sections. All authors contributed to the revisions of the manuscript and take public responsibility for its content.

## Pre-publication history

The pre-publication history for this paper can be accessed here:

http://www.biomedcentral.com/1471-2288/11/35/prepub
